# Supplementary material for: Suicidal ideation following self-reported COVID-19-like symptoms or serology-confirmed SARS-CoV-2 infection in France: A propensity score weighted analysis from a cohort study
Source: PLoS Med. 2023 Feb 14;20(2):e1004171. doi: 10.1371/journal.pmed.1004171 (PMC10072374; doi:10.1371/journal.pmed.1004171)
Supplement: S1 STROBE checklist — (DOCX) [file pmed.1004171.s001.docx]

STROBE Statement—Checklist of items that should be included in reports of ***cohort studies***

|  | Item No | Recommendation | Section name & paragraph number |
| --- | --- | --- | --- |
| **Title and abstract** | 1 | (*a*) Indicate the study’s design with a commonly used term in the title or the abstract | Title |
|  |  | (*b*) Provide in the abstract an informative and balanced summary of what was done and what was found | Abstract |
| Introduction | | | |
| Background/rationale | 2 | Explain the scientific background and rationale for the investigation being reported | Introduction, 1-3 |
| Objectives | 3 | State specific objectives, including any prespecified hypotheses | Introduction, 4 |
| Methods | | | |
| Study design | 4 | Present key elements of study design early in the paper | Methods, Study population, 1-3 |
| Setting | 5 | Describe the setting, locations, and relevant dates, including periods of recruitment, exposure, follow-up, and data collection | Methods, Study population, 1-3, Fig 1 |
| Participants | 6 | (*a*) Give the eligibility criteria, and the sources and methods of selection of participants. Describe methods of follow-up | Methods, Study population, 1-2 |
|  |  | (*b*) For matched studies, give matching criteria and number of exposed and unexposed |  |
| Variables | 7 | Clearly define all outcomes, exposures, predictors, potential confounders, and effect modifiers. Give diagnostic criteria, if applicable | Methods, outcome  Methods, exposures  Methods, propensity score covariates |
| Data sources/ measurement | 8* | For each variable of interest, give sources of data and details of methods of assessment (measurement). Describe comparability of assessment methods if there is more than one group | Methods, outcome  Methods, exposures  Methods, propensity score covariates |
| Bias | 9 | Describe any efforts to address potential sources of bias | / |
| Study size | 10 | Explain how the study size was arrived at | Methods, statistical analyses, 1 & Fig 2 |
| Quantitative variables | 11 | Explain how quantitative variables were handled in the analyses. If applicable, describe which groupings were chosen and why | Methods, exposure : serology-confirmed SARS-CoV-2 infection  Methods, propensity score covariates |
| Statistical methods | 12 | (*a*) Describe all statistical methods, including those used to control for confounding | Methods, statistical analyses, inverse probability weighting and modified Poisson regression models, 1 |
|  |  | (*b*) Describe any methods used to examine subgroups and interactions | Methods, statistical analyses, sensitivity analyses, 2-6 |
|  |  | (*c*) Explain how missing data were addressed | Methods, statistical analyses, sensitivity analyses, 7 |
|  |  | (*d*) If applicable, explain how loss to follow-up was addressed |  |
|  |  | (*e*) Describe any sensitivity analyses | Methods, statistical analyses, sensitivity analyses, 1-6 |
| Results | | |  |
| Participants | 13* | (a) Report numbers of individuals at each stage of study—eg numbers potentially eligible, examined for eligibility, confirmed eligible, included in the study, completing follow-up, and analysed | Methods, study population, 2 |
|  |  | (b) Give reasons for non-participation at each stage |  |
|  |  | (c) Consider use of a flow diagram | Methods, statistical analyses, Fig2 |
| Descriptive data | 14* | (a) Give characteristics of study participants (eg demographic, clinical, social) and information on exposures and potential confounders | Results, Main analysis, Table 1, 2 & 3 |
|  |  | (b) Indicate number of participants with missing data for each variable of interest | Methods, statistical analyses, Fig 2 |
|  |  | (c) Summarise follow-up time (eg, average and total amount) | Abstract – method section |
| Outcome data | 15* | Report numbers of outcome events or summary measures over time | NA |

| Main results | 16 | (*a*) Give unadjusted estimates and, if applicable, confounder-adjusted estimates and their precision (eg, 95% confidence interval). Make clear which confounders were adjusted for and why they were included | Results, main analysis, 1-2 |
| --- | --- | --- | --- |
|  |  | (*b*) Report category boundaries when continuous variables were categorized |  |
|  |  | (*c*) If relevant, consider translating estimates of relative risk into absolute risk for a meaningful time period |  |
| Other analyses | 17 | Report other analyses done—eg analyses of subgroups and interactions, and sensitivity analyses | Results, sensitivity analyses, 1-5 |
| Discussion | | | |
| Key results | 18 | Summarise key results with reference to study objectives | Discussion, summary of findings |
| Limitations | 19 | Discuss limitations of the study, taking into account sources of potential bias or imprecision. Discuss both direction and magnitude of any potential bias | Discussion, strengths and limitations, 1-4 |
| Interpretation | 20 | Give a cautious overall interpretation of results considering objectives, limitations, multiplicity of analyses, results from similar studies, and other relevant evidence | Discussion, serology-confirmed SARS-CoV-2 infection and suicidal ideation  Discussion self-reported COVID-19 like symptoms |
| Generalisability | 21 | Discuss the generalisability (external validity) of the study results | Discussion, strengths and limitation, 4 |
| Other information | | | |
| Funding | 22 | Give the source of funding and the role of the funders for the present study and, if applicable, for the original study on which the present article is based | NA (on journal website) |

*Give information separately for exposed and unexposed groups.

**Note:** An Explanation and Elaboration article discusses each checklist item and gives methodological background and published examples of transparent reporting. The STROBE checklist is best used in conjunction with this article (freely available on the Web sites of PLoS Medicine at http://www.plosmedicine.org/, Annals of Internal Medicine at http://www.annals.org/, and Epidemiology at http://www.epidem.com/). Information on the STROBE Initiative is available at http://www.strobe-statement.org.
