# Supplementary material for: Suicidal ideation following self-reported COVID-19-like symptoms or serology-confirmed SARS-CoV-2 infection in France: A propensity score weighted analysis from a cohort study
Source: PLoS Med. 2023 Feb 14;20(2):e1004171. doi: 10.1371/journal.pmed.1004171 (PMC10072374; doi:10.1371/journal.pmed.1004171)
Supplement: S1 Acknowledgements — (DOCX) [file pmed.1004171.s002.docx]

Suicidal ideation following self-reported COVID-19 like symptoms or serology-confirmed SARS-CoV-2 infection in France: a propensity score weighted analysis from a cohort study.

***S1 Acknowledgments***

The EpiCoV study group: Josiane Warszawski (co-principal investigator) and Nathalie Bajos (co-principal investigator), Guillaume Bagein, François Beck, Emilie Counil, Florence Jusot, Nathalie Lydie, Claude Martin, Laurence Meyer, Philippe Raynaud, Alexandra Rouquette, Ariane Pailhé, Delphine Rahib, Patrick Sillard, Rémy Slama, Alexis Spire.

Lead : Josiane Warszawski, INSERM CESP U1018, AP-HP Epidemiology and Public Health Service, Université Paris-Saclay, Le Kremlin-Bicêtre, France

Nathalie Bajos (co-lead), Iris–Institut de Recherche Interdisciplinaire sur les enjeux sociaux, Inserm, Aubervilliers, France ; Ecole des Hautes Etudes en Sciences Sociales, Paris, France

Guillaume Bagein, DREES—Direction de la Recherche, des Etudes, de l’évaluation et des statistiques, Paris, France

François Beck, Santé Publique France, Saint-Maurice France

Emilie Counil, French Institute for Demographic Studies (INED), France

Florence Jusot, Université Paris Dauphine, Paris, France

Nathalie Lydie, Santé Publique France, Saint-Maurice France

Claude Martin, ARENES UMR 6051, CNRS, EHESP, Rennes, France

Laurence Meyer, INSERM CESP U1018, AP-HP Epidemiology and Public Health Service,

Université Paris-Saclay, Le Kremlin-Bicêtre, France

Philippe Raynaud, DREES—Direction de la Recherche, des Etudes, de l’évaluation et des statistiques, Paris, France

Alexandra Rouquette, INSERM CESP U1018, AP-HP Epidemiology and Public Health Service, Université Paris-Saclay, Le Kremlin-Bicêtre, France

Ariane Pailhé, French Institute for Demographic Studies (INED), France

Delphine Rahib, Santé Publique France, Saint-Maurice France

Patrick Sillard, Institut National de la statistique et des études économiques, Montrouge, France

Rémy Slama, Institut thématique de Santé Publique, INSERM, Paris France, Inserm, CNRS, Team of Environmental Epidemiology applied to Reproduction and Respiratory Health, Institute for Advanced Biosciences, University Grenoble Alpes, Grenoble, France

Alexis Spire, Iris–Institut de Recherche Interdisciplinaire sur les enjeux sociaux, Inserm, Aubervilliers, France

We sincerely thank all the participants in the EpiCoV study.

We warmly thank the INSERM staff, including, in particular, Carmen Calandra, Karim Ammour, Jean-Marc Boivent, Jean-Marie Gagliolo, Frédérique Le Saulnier, and Frédéric Robergeau, who worked with considerable dedication and commitment to make it possible to develop, in record time, and to maintain all regulatory, budgetary, technical, and logistical aspects of the EpiCov study.

We warmly thank the staff of Santé publique France, and especially Lucie Duchesne, who played a major role in organization and quality assurance for the seroprevalence component of the EpiCov study.

We thank the CRB biobanks staff, and especially their heads, Dr Isabelle Pellegrin, and Julien Jeanpetit (Centre Hospitalier Universitaire Robert Pellegrin, Bordeaux, France), Pr Edouard Tuaillon Centre de Ressources Biologiques du CHU de Montpellier), Dr Yves-Edouard Herpe (Centre de Ressources Biologiques Biobanque de Picardie), Pr Jacqueline Deloumeaux (Centre biologique du CHU de la Guadeloupe), Dr Rémi Neviere (CeRBiM, Centre de Ressources Biologiques de la Martinique), Julien Eperonnier, Estelle Nobecourt (Centre de Ressources Biologiques de la Réunion) for the quality of DBS sample management of the EpiCov study. We thank the biobank team in Inserm SC10, particularly Sophie Circosta. We also thank the staff of the UVE virology department, for the high-quality management of such a large number of serological assays.

We thank the staff of DREES and INSEE, for their collaboration in the implementation of the study, methodological input, sample selection, and the complex development of weights to correct for non-response.

We thank the Ipsos staff, including Christophe David and Valérie Blineau in particular, for their major contribution to the quality of data collection.
