# Supplementary material for: Suicidal ideation following self-reported COVID-19-like symptoms or serology-confirmed SARS-CoV-2 infection in France: A propensity score weighted analysis from a cohort study
Source: PLoS Med. 2023 Feb 14;20(2):e1004171. doi: 10.1371/journal.pmed.1004171 (PMC10072374; doi:10.1371/journal.pmed.1004171)
Supplement: S1 Supporting information — (DOCX) [file pmed.1004171.s003.docx]

Suicidal ideation following self-reported COVID-19 like symptoms or serology-confirmed SARS-CoV-2 infection in France: a propensity score weighted analysis from a cohort study.

***S1 Supporting information: Detailed description of the pre-pandemic mental health disorders variable construction***

At the second follow-up in Summer 2021, participants were asked if a physician ever told them they had a mental health or substance use disorder. If so, they were asked to report which one and when (before the pandemic vs since the pandemic) from a list. The list included anxiety disorders, mood disorders, bipolar disorders, eating disorders, personality disorders, substance use disorders and schizophrenia. Participants reporting a physician ever telling them they had at least one of these disorders before the pandemic were included in the pre-pandemic mental health disorder group.

At the first follow-up in Autumn 2020, participants were asked to report life-course suicide attempt and if so, the period of the last one, i.e. before Autumn 2019, between Autumn 2019 and 17^th^ of March 2020, between 17^th^ of March and the 11^th^ of May 2020 (first lockdown), between 12^th^ of May 2020 and Autumn 2020. Participants reporting a last suicide attempt before Autumn 2019 were included in the pre-pandemic mental disorder group.

At the second follow-up in Summer 2021, participants were asked if a physician ever told them they had a mental health or substance use disorder. If so, they were asked to report which one and when (before the pandemic vs since the pandemic) from a list. The list included anxiety disorders, mood disorders, bipolar disorders, eating disorders, personality disorders, substance use disorders and schizophrenia. Participants reporting a physician ever telling them they had at least one of these disorders before the pandemic were included in the pre-pandemic mental health disorder group.
