# Supplementary material for: Suicidal ideation following self-reported COVID-19-like symptoms or serology-confirmed SARS-CoV-2 infection in France: A propensity score weighted analysis from a cohort study
Source: PLoS Med. 2023 Feb 14;20(2):e1004171. doi: 10.1371/journal.pmed.1004171 (PMC10072374; doi:10.1371/journal.pmed.1004171)
Supplement: S2 Supporting information — (DOCX) [file pmed.1004171.s004.docx]

Suicidal ideation following self-reported COVID-19 like symptoms or serology-confirmed SARS-CoV-2 infection in France: a propensity score weighted analysis from a cohort study.

## S2 Supporting information: Evolution of total number of deaths by French departments during the first COVID-19 epidemic wave.

The first and deadliest COVID-19 epidemic wave occurred during the early Spring 2020 in France. At the end of April 2020, 238,271 deaths had been recorded since the beginning of 2020. In 2019 and 2018 respectively, the number of deaths recorded for the same period were 219,041 and 222,828 (1). At the end of May 2021, 291,915 deaths had been recorded, 9% more than in 2019 (2). Between 01/03/2020 and 30/04/2020, the most intense phase of the epidemic, about 26% and 17% more deaths were recorded, compared to 2019 and 2018 respectively.

The three regions with the highest rise in cumulated deaths were the Ile-de-France (89% increase), the Grand Est (54% increase) and the Hauts-de-France regions (27%). Compared to March-April 2019, all of the eight departments of the Ile-de-France region saw an increase in cumulated deaths of more than 40% (from 68.4% in Paris to 123.4% in Seine-Saint-Denis). For the Grand Est region, four of the ten departments also had an increase of more than 40% in cumulated deaths (from 56.5% in Bas-Rhin to 115.9% in Haut-Rhin), compared to 2019. The other departments ranged between 14.3% and 38.9%. For the Hauts-de-France region, two departments had an increase of more than 40% (50.4% in Aisne, 60% in Oise) while the other three ranged between 11.3% and 26.4% (1).

***Reference***

1. Institut National de la Statistique et des Etudes Economiques I. Evolution of deaths between 1 March and 30 April 2020 France2020 [updated 28/10/2022. Evolution of deaths between 1 March and 30 april 2020, i.e. France's first COVID-19 epidemic wave]. Available from: <https://www.insee.fr/en/statistiques/4504742?sommaire=4493845>.

2. Institut National de la Statistique et des Etudes Economiques I. Évolution du nombre de décès du 1ᵉʳ janvier au 31 mai 2021 France2021 [updated 02/07/2021. Available from: <https://www.insee.fr/fr/statistiques/5404610?sommaire=4487854>.

3. VanderWeele TJ, Ding P. Sensitivity Analysis in Observational Research: Introducing the E-Value. Ann Intern Med. 2017;167(4):268-74.
