# Supplementary material for: Suicidal ideation following self-reported COVID-19-like symptoms or serology-confirmed SARS-CoV-2 infection in France: A propensity score weighted analysis from a cohort study
Source: PLoS Med. 2023 Feb 14;20(2):e1004171. doi: 10.1371/journal.pmed.1004171 (PMC10072374; doi:10.1371/journal.pmed.1004171)

Suicidal ideation following self-reported COVID-19 like symptoms or serology-confirmed SARS-CoV-2 infection in France: a propensity score weighted analysis from a cohort study.

## S3 Supporting information: Directed Acyclic Graph (DAG)

A DAG helps visualizing the different pathways through which the exposure, outcome and covariates are related to each other. It also gives the minimum adjustment required to assess the direct relationship between the exposure and outcome, based on the specified pathways and covariates. For the present study, we only used a DAG for visualization as we decided to base our covariate selection on current available literature.

*S3 figure: directed acyclic graph of the links between COVID-19 disease, suicidal ideation and all identified covariates*


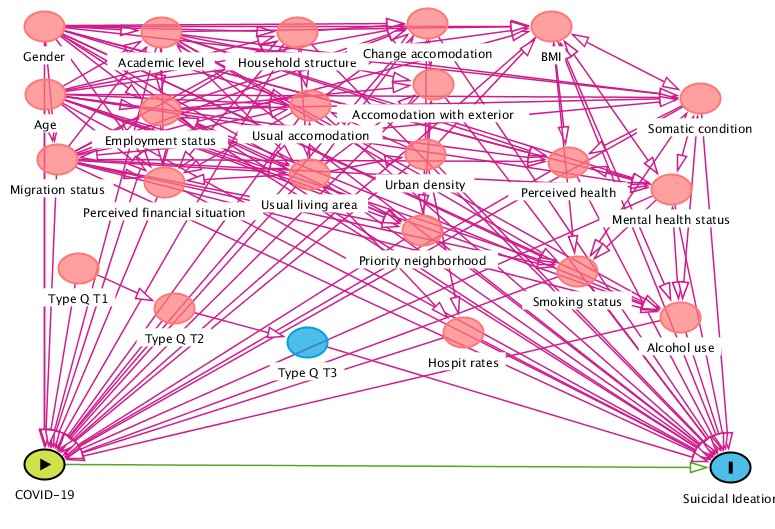

Supplement: S3 Supporting information — (DOCX) [file pmed.1004171.s005.docx]
