# Supplementary material for: Suicidal ideation following self-reported COVID-19-like symptoms or serology-confirmed SARS-CoV-2 infection in France: A propensity score weighted analysis from a cohort study
Source: PLoS Med. 2023 Feb 14;20(2):e1004171. doi: 10.1371/journal.pmed.1004171 (PMC10072374; doi:10.1371/journal.pmed.1004171)

Suicidal ideation following self-reported COVID-19 like symptoms or serology-confirmed SARS-CoV-2 infection in France: a propensity score weighted analysis from a cohort study.

## S4 supporting information: multiple imputation

We used raw covariates as much as possible, with “do not wish to answer” and “do not know” modalities when applicable. 24 covariates were used in the multiple imputation model, with 13 of them having up to 4.71% missing data. The seed was randomly defined as 91118. Below is a screen capture of the SAS code used. The matching covariate definitions can be found in the supplementary tables file in S6 table. Number of individuals with missing data in each covariate can be found in the supplementary tables file in S2 table.

*S4 figure: screen capture of the SAS code used for imputation analyses*


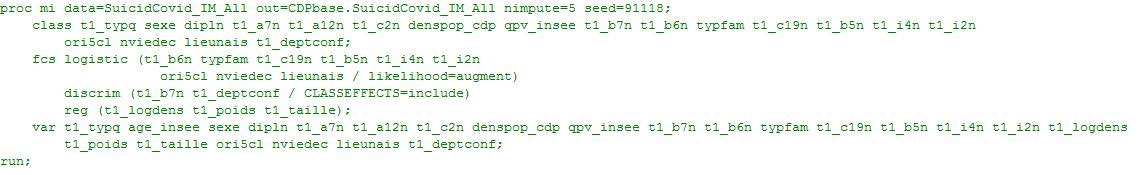

Supplement: S4 Supporting information — (DOCX) [file pmed.1004171.s006.docx]
