# Supplementary material for: Suicidal ideation following self-reported COVID-19-like symptoms or serology-confirmed SARS-CoV-2 infection in France: A propensity score weighted analysis from a cohort study
Source: PLoS Med. 2023 Feb 14;20(2):e1004171. doi: 10.1371/journal.pmed.1004171 (PMC10072374; doi:10.1371/journal.pmed.1004171)
Supplement: S5 Supporting information — (DOCX) [file pmed.1004171.s007.docx]

Suicidal ideation following self-reported COVID-19 like symptoms or serology-confirmed SARS-CoV-2 infection in France: a propensity score weighted analysis from a cohort study.

***S5 supporting information: propensity score***

**Inverse Probability weighting (IPW) method**

In the present work, IPW was used as an adjustment strategy to assess the direct association of two binary COVID-19 exposures in 2020 with subsequent suicidal ideation in 2021. The two COVID-19 exposures were separately used, self-reported COVID-19 symptoms (yes or no) and serology-confirmed SARS-CoV-2 infection (yes or no). Participants reporting COVID-19 symptoms or with a serology -confirmed SARS-CoV-2 infection will be referred to as exposed below. First, propensity scores, i.e. probabilities (p), of either self-reporting COVID-19 symptoms or having serology-confirmed SARS-CoV-2 infection were computed using a logistic regression where the COVID-19 exposures were explained by the covariates listed in the manuscript. Then inverse probability weights were calculated as follow:

- In COVID-19 exposed group: ipw = $\frac{1}{p}$
- In non-exposed group: ipw = $\frac{1}{\left( 1-p \right)}$

Where p is the probability of self-reporting symptoms, or having a serology-confirmed SARS-CoV-2 infection, according to the covariates.

IPW is an average treatment effect methodology where the weights are used to create a subpopulation were both the exposed and non-exposed groups have the same covariate distributions as the whole population.

**Average treatment effect on the treated method**

To test robustness of our results we assessed what would have happened to exposed participants if they had not been exposed. To do so, we weighted the non-exposed group to have the same covariate distributions as the exposed group. Conservation of results as compare to IPW method increases the reliability of the results. As for ipw, propensity scores were used to calculate relevant weights:

- In exposed group: watt = 1
- In non-exposed group: watt = $\frac{p}{(1-p)}$

Where p is the probability of self-reporting symptoms, or having a serology-confirmed SARS-CoV-2 infection, according to the covariates.

**Covariates used for propensity scores calculation**

All covariates listed in the manuscript were used to compute the scores. Age was squared and used as a continuous covariate.

Parameters for propensity score calculation are available in supplementary tables S7 Table and S8 Table for COVID-19 like symptoms, and S9 Table and S10 Table for SARS-CoV-2 serology.
