# Supplementary material for: Suicidal ideation following self-reported COVID-19-like symptoms or serology-confirmed SARS-CoV-2 infection in France: A propensity score weighted analysis from a cohort study
Source: PLoS Med. 2023 Feb 14;20(2):e1004171. doi: 10.1371/journal.pmed.1004171 (PMC10072374; doi:10.1371/journal.pmed.1004171)
Supplement: S6 Supporting information — (DOCX) [file pmed.1004171.s008.docx]

Suicidal ideation following self-reported COVID-19 like symptoms or serology-confirmed SARS-CoV-2 infection in France: a propensity score weighted analysis from a cohort study.

***S6 Supporting information: graphic representation of sensitivity analyses results***

S5 Figure: relative risk of self-reported COVID-19 like symptoms in 2020, in various samples and subgroups, with later suicidal ideation in 2021, using inverse probability weighting and modified Poisson regression models, results from the French EpiCov study
