# Supplementary material for: Suicidal ideation following self-reported COVID-19-like symptoms or serology-confirmed SARS-CoV-2 infection in France: A propensity score weighted analysis from a cohort study
Source: PLoS Med. 2023 Feb 14;20(2):e1004171. doi: 10.1371/journal.pmed.1004171 (PMC10072374; doi:10.1371/journal.pmed.1004171)
Supplement: Attachment — Submitted filename: Response to reviewer.docx [file pmed.1004171.s019.docx]

Camille Davisse-Paturet, on behalf of all co-authors
CESP-INSERM U1018
Equipe Psychiatrie du Développement et trajectoires,
97 boulevard de Port-Royal,
75014 Paris, France
[Camille.davisse-paturet@inserm.fr](mailto:Camille.davisse-paturet@inserm.fr)
+33158412848

21 October, 2022

Dear Reviewers,

We thank you for the time you took to thoroughly read and comment our work. We believe your comments significantly improved the manuscript, especially in terms of clarity and completeness. The supportive tone of some comments was really appreciated.

Below is our point by point response to each comment, the changes made and where to find them in the revised version.

We hope that you will find our revision satisfactory and remain at your disposal if needed.

Respectfully,

Camille Davisse-Paturet, on behalf of all co-authors

**Comments from the Academic Editor**

**1) First I have some logical difficulty in understanding the examined cause-effect relationship. The outcome is the suicidal ideation between Dec 2020 and June 2021, based on a single question “Have you thought about committing suicide in the past year” (and then excluding those who said they did so in Nov 2020). The exposure is self-reported COVID-19 symptoms or serology-confirmed SARS-COV2 infection at the 2nd wave, hence between the beginning of the pandemic and Nov 2020.**

**Can one expect to have an increase (or decrease) in thinking about committing suicide in, say, May 2021 when they had COVID-19 symptoms or SARS-COV2 infection in May 2020? If any such cause-effect is present, would it not be more natural to expect such in June-November 2021 but how are such possibilities accounted for or not accounted for in the present analyses?**

*Response:*

Thank you for this comment. We revised our manuscript to clarify this aspect.

Regarding suicidal ideation in 2021, no information was asked to participants regarding the exact timing of occurrence or persistence of this ideation. Regarding COVID-19 like symptoms in 2020, participants were asked to report the month of the last occurrence. We thus decided to remove participants reporting suicidal ideation before December 2020 to clarify, with this design, temporality between exposure (before December 2020) and outcome (after December 2020)

Page 8 line 166-180: “Suicidal ideation in 2020, as well as life course suicide attempts were assessed at the first follow-up in Autumn 2020 (Fig 1). The related questions were respectively: “In the last 12 months, have you thought about “killing yourself by suicide”?” and “In your lifetime, have you ever attempted suicide? If so, when was the last one?”. Yet, mediators are not supposed to be included in propensity score calculation. Suicidal ideation and suicide attempts in 2020 could act as mediator in the association of self-reported COVID-19 like symptoms in 2020 and serology-confirmed SARS-CoV2 infection in 2020 with suicidal ideation in 2021. But, in EpiCov, no data were available regarding the first occurrence of COVID-19 like symptoms in 2020 or date of SARS-CoV2 infection in 2020. We could therefore not ascertained if suicidal ideation or suicide attempt in 2020 occurred before or after the two COVID-19 exposures. To ensure no mediator was included in propensity score calculation, participants reporting suicidal ideation or a history of suicide attempt in 2020 or who did not provide information on the timing of their last suicide attempt, had to be removed from the statistical analyses. Participants who reported a last suicide attempt before 2020 were included in the pre-pandemic mental health disorder covariate.”

However, you are right that we did not take into account the time between the occurrence of COVID-19 like symptoms and subsequent occurrence of suicidal ideation in the main analyses. Following your comment, we thus decided to perform sensitivity analyses to evaluate if the association of self-reported COVID-19 like symptoms with subsequent suicidal ideation was different according to the time of the last onset of COVID-19 like symptoms in 2020, keeping in mind that we do not have information on the time of occurrence of suicidal ideation in 2021.

Among participants who reported COVID-19 like symptoms in 2020:

- 27.2% [25.9 – 28.6] reported a last onset in the two months before first follow-up,
- 25.8% [24.5 – 27.1] reported a last onset four to six months before first follow-up,
- 47.0% [45.4 – 48.5] reported a last onset more than six months before first follow-up.

Three sensitivity analyses were thus performed using propensity scores of the following exposures:

1) Self-reported COVID-19 like symptoms with last onset in the two months before first follow-up (yes vs no COVID-19 symptoms reported)
2) Self-reported COVID-19 like symptoms with last onset four to six months before first follow-up (yes vs no COVID-19 symptoms reported)
3) Self-reported COVID-19 like symptoms with last onset more than six months before first follow-up (yes vs no COVID-19 symptoms reported)

In line with main analyses, self-reported COVID-19 like symptoms with last onset in the two months before first follow-up were associated with a higher risk of subsequent suicidal ideation in 2021 (Relative risk [95% confidence interval]: 1.82 [1.39 – 2.39]). However, when the last onset of self-reported COVID-19 like symptoms was four to six months or more than six months before first follow-up, no association was found with suicidal ideation in 2021 (RR [95%CI]: 1.24 [0.91 – 1.67] and 1.24 [0.95 – 1.62] respectively).

These sensitivity analyses were added in the revised version of the manuscript.

Page 10 Line 229-241: “Time between self-reported COVID-19 like symptoms and suicidal ideation was also explored. Among participants who reported COVID-19 like symptoms in 2020, 27.2% [25.9 – 28.6] reported a last onset in the two months before first follow-up in Autumn 2020, 25.8% [24.5 – 27.1] reported a last onset four to six months before first follow-up in Autumn 2020, and 47.0% [45.4 – 48.5] reported a last onset more than six months before first follow-up in Autumn 2020. 19 participants had missing data on timing of the last COVID-19 symptoms onset. We conducted three sensitivity analyses ascertaining the association of self-reported COVID-19 like symptoms in 2020 with suicidal ideation in 2021, where COVID-19 symptoms were defined as follow: 1) Self-reported COVID-19 like symptoms with last onset in the two months before first follow-up in Autumn 2020; 2) self-reported COVID-19 like symptoms with last onset four to six months before first follow-up in Autumn 2020; 3) self-reported COVID-19 like symptoms with last onset more than six months before first follow-up in Autumn 2020. In all three sensibility analyses, the reference group reported no COVID-19 like symptoms.”

Page 11 Line 280-290: “Regarding the sensitivity analyses ascertaining accounting for the period of the last symptom onset, in line with main analyses, self-reported COVID-19 like symptoms with last onset in the two months before first follow-up were associated with a higher risk of subsequent suicidal ideation in 2021 (Relative risk [95% confidence interval]: 1.82 [1.39 – 2.39]). However, when the last onset of self-reported COVID-19 like symptoms was four to six months or more than six months before first follow-up, no association was found with suicidal ideation in 2021 (RR [95%CI]: 1.24 [0.91 – 1.67] and 1.24 [0.95 – 1.62] respectively).”

**In a similar vein, if someone had symptoms or infection in Jan 2021, it could have had an effect on suicidal ideation between Jan and June 2021 (this is a hypothesis that the study is examining), but where are such possibilities accounted for in the present analyses?**

*Response*:

Indeed. The first version of our manuscript did not account for this, so we performed another sensitivity analysis, removing participants who reported COVID-19 like symptoms since December 2020 at the second follow-up in Summer 2021 (n=4,564). In line with main analyses, self-reported COVID-19 like symptoms in 2020 were associated to subsequent suicidal ideation in 2021 (RR [95%CI]: 1.40 [1.14 – 1.71]). This analysis has been added in the revised version of the manuscript.

Page 10 Line 224-226: “As occurrence of COVID-19 like symptoms since December 2020, ascertained at the second follow-up in Summer 2021, can be associated with suicidal ideation between December 2020 and Summer 2021, a sensibility analysis (i.e., propensity score calculation and IPWeighted Poisson regression model) was performed, removing participants who reported COVID-19 like symptoms between December 2020 and Summer 2021 (n=4,564).”

Page 11 Line 277-279: “In line with main analyses, among participants who reported no COVID-19 like symptoms between December 2020 and Summer 2021, self-reported COVID-19 like symptoms in 2020 were associated to a higher risk of suicidal ideation in 2021 (RRipw: 1.40 [1.14 – 1.71]).”

**2) As suggested by Reviewer #3, I think the timeline of the study is essential to understanding all these and an expanded version of their Supplementary Fig 1 must be included in the main text.**

*Response:*

As suggested, the supplementary Fig 1 is now included in the main text under the name Fig 1.

**3) Like Reviewer #2, I also have a difficulty calling this study as nationally representative, when only 16% of the originally nationally representative population is analyzed.**

*Response:*

We agree that our study is not representative. By nationwide, we meant that data were collected across the entire country, including oversea territories. The term nationwide was nonetheless removed from the revised manuscript to minimize misunderstanding. Of note, descriptive statistics can be considered as representative of the general population as they were weighted by study weights.

**4) Looking at their Supplementary Fig 1, the variables (pre-pandemic diagnoses measured at the 3rd wave) are included in calculating the IPW. Sure, they are supposed to be pre-baseline but they are subjectively recalled at the same time as the outcome measurement. I wonder if the authors need not report at least a sensitivity analysis excluding this variable in the IPW calculation.**

*Response:*

We agree, a memory bias cannot be ruled out regarding this variable and therefore performed the suggested sensitivity analysis. Results were in line with main analyses with self-reported COVID-19 like symptoms in 2020 associated to subsequent suicidal ideation in 2021 (RR [95%CI]: 1.51 [1.27 – 1.79]) while serology-confirmed SARS-CoV2 infection was not (RR [95%CI]: 0.87 [0.69 – 1.11]).

Page 9 Line 221-223: “As pre-pandemic mental health disorder were partly assessed at the second follow-up, a memory bias cannot be ruled out. A sensitivity analyses removing the variable from propensity scores calculation was therefore performed.”

Page 11-Line 273-276: “When removing the pre-pandemic mental health disorder variable from propensity scores calculation, self-reported COVID-19 like symptoms in 2020 were associated to subsequent suicidal ideation in 2021 (RR [95%CI]: 1.51 [1.27 – 1.79]) while serology-confirmed SARS-CoV2 infections were not (RR [95%CI]: 0.87 [0.69 – 1.11]).”

**5) Although the statistical reviewer is basically OK with the analyses, I am not sure if the authors have done their best to impute the missing data. When the authors can use multiple imputation, is it justified to exclude almost 30000 (so amounting to some 60% of the final sample) for missing that variable (while they must have had other data because these 30000 did respond in principle to the surveys). Likewise some 2400 people are excluded because they had missing data on suicidality (but apparently had other data): in this situation, could they also not impute the missing outcome, using some auxiliary variables?**

*Response:*

We understand the high number of participants removed from analyses can decrease generalizability and have added this a limit in the discussion section. Although we imputed missing data on covariates, there are debates in the literature about whether the imputation of exposure and outcomes is methodologically sound (1-4). Regarding the present study, we decided not to impute the outcome and both exposures as assuming data were missing at random for suicidal ideation, COVID-19 like symptoms and participation to serology testing seemed questionable. The prevalence of suicidal ideation did not statistically differed between the 2,051 participants with missing data regarding self-reported COVID-19 like symptoms in 2020 and the ones who answered (chi² test, 2.7% of participants with missing symptoms reported suicidal ideation vs 2.8% in participants with a COVID-19 like symptoms data, p=0.84). The same pattern was observed for serology-confirmed SARS-CoV2 infection, although the difference was closer to statistical significance (chi² test, 2.7% of participants with missing serology reported suicidal ideation vs 2.9% in participants with serology data, p=0.058). Exclusion of participants with no serology data could have lead to overestimate the association of serology-confirmed SARS-CoV2 infection in 2020 with subsequent suicidal ideation. Considering we did not found a significant association and the reliability of our results, we hypothesized the exclusion of participants with missing data on the outcome and both exposures had limited effect on our results.

Abstract – method section: “Limitations of this study include the use of a single question to assess suicidal ideation, the use of self-reported history of mental health disorders, and limited generalizability due to attrition bias.”

Page 14 Line 381-387: “Lastly, although the EpiCov cohort is based on a randomly selected sample, attrition overtime as well as successive steps of participant selection for analyses purpose limit the generalizability of our results. Difference between included and excluded participants can be found in supplementary table 1. Of note, included participants were less likely to report suicidal ideation in 2021 but more likely to report COVID-19 like symptoms, potentially leading to an underestimation of the association between the two. Such difference were however not found for serology-confirmed SARS-CoV2 infection.”

**6) I agree with Reviewer #3 that the relationship (overlaps) between those with self-reported COVID-19 symptoms and those with serologically confirmed SARS-COV2 infection. Could they be non-overlapping? If so, what could each mean, especially the former? Could some further analysis focusing on those with self-reported symptoms but without serological confirmation shed some more light?**

*Response*:

This is a very relevant question that we investigated in ascertaining the association of self-reported COVID-19 like symptoms before December 2020 with subsequent suicidal ideation in 2021, separately in participants with a positive SARS-CoV2 serology (RR [95%CI]: 1.49 [0.95 – 2.32]) and in participants whose SARS-CoV2 serology was negative (RR [95%CI]: 1.49 [1.22 – 1.83]). The RR was of similar value but a lack of statistical power may have resulted in a non-significant result in participants with a positive SARS-CoV2 serology as only 48 of them reported both COVID-19 like symptoms before December 2020 and subsequent suicidal ideation.

Page 10 Line242-245: “Lastly, to explore the intersection of both COVID-19 exposures with respect to suicidal ideation, association of self-reported COVID-19 like symptoms before December 2020 with subsequent suicidal ideation in 2021 was separately assessed according the SARS-CoV2 serological status.”

Page 11 Line 287-291: “Lastly, in the serology-confirmed SARS-CoV2 infection group, self-reported COVID-19 like symptoms in 2020 were not associated with suicidal ideation in 2021 (RR_ipw_: 1.49 [0.95 – 2.32]), while in the negative serology group, self-reported COVID-19 like symptoms before December 2020 were associated to a higher risk of subsequent suicidal ideation in 2021 (RR_ipw_: 1.49 [1.22 – 1.83]).”

Moreover, as requested by Reviewer #3, descriptive analyses of sociodemographic and health-related characteristics according to self-reported COVID-19 like symptoms and serology-confirmed SARS-CoV2 infection were added in supplementary material (Tables 2and 3).

**Requests from the editor**

**Throughout: Consider whether the term ‘COVID-19 illness’ ought to be updated to ‘COVID-19 symptoms’ to describe more accurately what was assessed in the study.**

*Response*:

We made the requested changes.

**Title: Related to the above, please revise your title to “COVID-19 symptoms, SARS-CoV2 infection, and suicidal ideation in France: A population-based propensity score–matched cohort study”, or similar.**

*Response*:

The title has been rephrased accordingly and reads: “Suicidal ideation following self-reported COVID-19 like symptoms or serology-confirmed SARS-CoV2 infection in France: a propensity score weighted analysis from a cohort study.” We did not include the score-matched suggestion as propensity scores were used for inverse probability weighting and weighting on the treated, not for matching.

**Please structure your abstract using the PLOS Medicine headings (Background, Methods and Findings, Conclusions).**

*Response*:

The requested changes were made.

**Abstract Methods and Findings:**

*** Please include the length of follow up.**

*Response*:

The median lengths of follow-up (13.7 months) was added in the first paragraph if abstract’s methods section.

Abstract – method section “52,050 participants from the French population-based EpiCov cohort were included (median follow-up time = 13.7 months).”

*** In the last sentence of the Abstract Methods and Findings section, please describe the main limitations of the study's methodology.**

*Response*:

The main limitations of the study were added at the end of the abstract’s methods section.

Abstract method section: “Limitations of this study include the use of a single question to assess suicidal ideation, the use of self-reported history of mental health disorders, and limited generalizability due to attrition bias.”

**The Data Availability Statement (DAS) requires revision. If the data are owned by a third party but freely available upon request, please note this and state the owner of the data set and contact information for data requests (web or email address). Note that a study author cannot be the contact person for the data.**

**At this stage, we ask that you include a short, non-technical Author Summary of your research to make findings accessible to a wide audience that includes both scientists and non-scientists. The Author Summary should immediately follow the Abstract in your revised manuscript. This text is subject to editorial change and should be distinct from the scientific abstract. Please see our author guidelines for more information: https://journals.plos.org/plosmedicine/s/revising-your-manuscript#loc-author-summary**

*Response*:

The following non-technical author summary was added right after the abstract section.

Line 1-16: “Why was this study done?

This study was done to explore if COVID-19 like symptoms in 2020, as reported by participants, and SARS-CoV2 infection, as confirmed by serological tests, were associated to a higher risk of suicidal ideation in 2021.

What did the researchers do and find?

Using data from 52,050 participants followed three times between May 2020 and Summer 2021, we found that participants who reported COVID-19 like symptoms in 2020, but not those with a serologically confirmed SARS-CoV2 infection, were at higher risk of reporting suicidal ideation in 2021. These results account for sociodemographic and health-related factors.

What do these findings mean?

These findings suggest that individuals experiencing COVID-19 like symptoms in the first year of the pandemic, even with no serological confirmation of the infection, were at higher risk of later suicidal ideation. From a public health perspective, short communication on what to do when someone is experiencing distress, whether directly after the onset of symptoms or a few months later, may be beneficial to address increase in suicidal ideation.”

**Citations should be preceding punctuation.**

*Response*:

The requested changes were made.

**Please include paragraphs in your Introduction.**

*Response*:

The introduction section has been reorganized as suggested.

**The terms gender and sex are not interchangeable (as discussed in https://www.who.int/health-topics/gender); please use the appropriate term.**

*Response*:

The question used in EpiCov was “Are you…? A Man; A Woman”, which captures the self-evaluation of individual identity rather than the sex (male, female) attributed at birth. We believe this to be closer to the WHO’s definition of gender but cannot guarantee that all participants in EpiCov understood the question this way. Nonetheless, the term gender was used throughout the manuscript and in supplementary materials. The term “sexe” which can be found in supplementary material is the name of the gender variable used in our models. We acknowledge we could have chosen a more appropriate term.

**Ethics: Please specify whether informed consent was written or oral.**

*Response*:

As approved by relevant committees, participants were informed of the EpiCov study’s purpose, the data collection methods, the categories of data collected, the free and voluntary nature of participation in the study, the contact details of the data processors, the length of time the data will be kept, as well as the methods for exercising their rights through an information letter send by post, as well as email and shorter SMS when available. The letter was also available on the study website. Considering the full active information process, responding to the questionnaire was considered to be the expression of an express consent. Similar process was used at first and second follow-up.

**Please remove the Role of the Funding Source section.**

*Response*:

The aforementioned section was removed.

**Please remove the Authors contribution and Data Sharing, and Acknowledgements section from the main text as all the relevant information is captured as metadata via the Submission Form. You are welcome to still include an Acknowledgements section but it should not contain details of study funders.**

*Response*:

The aforementioned section were removed.

**Please remove italic formatting in the References and include the first six author names before et al.**

*Response*:

The requested changes were made. The Plos Medicine Endnote reference style format was applied to all references.

**Please do not report P<0.0001, report instead as P<0.001 (Supporting Information).**

*Response*:

The requested changes have been made.

**Please ensure that the study is reported according to the STROBE guideline, and include the completed STROBE checklist as Supporting Information. Please add the following statement, or similar, to the Methods: "This study is reported as per the Strengthening the Reporting of Observational Studies in Epidemiology (STROBE) guideline (S1 Checklist)."**

**The STROBE guideline can be found here: http://www.equator-network.org/reporting-guidelines/strobe/**

**When completing the checklist, please use section and paragraph numbers, rather than page numbers.**

**Did your study have a prospective protocol or analysis plan? Please state this (either way) early in the Methods section.**

**a) If a prospective analysis plan (from your funding proposal, IRB or other ethics committee submission, study protocol, or other planning document written before analyzing the data) was used in designing the study, please include the relevant prospectively written document with your revised manuscript as a Supporting Information file to be published alongside your study, and cite it in the Methods section. A legend for this file should be included at the end of your manuscript.**

**b) If no such document exists, please make sure that the Methods section transparently describes when analyses were planned, and when/why any data-driven changes to analyses took place.**

**c) In either case, changes in the analysis-- including those made in response to peer review comments-- should be identified as such in the Methods section of the paper, with rationale.**

The present study does not have a registered prospective protocol. An unpublished, informal analysis plan was made and discussed among study authors prior to the implementation of statistical analyses

Page 6-Line 86-88: “The present study does not have a registered prospective protocol. An unpublished, informal analysis plan was made and discussed among study authors prior to the implementation of statistical analyses.”

Page 9 Line 219-220: “In response to peer review comments, the following four sets of sensitivity analyses were also performed.”

**Comments from the reviewers:**

**Reviewer #1: Alex McConnachie, Statistical Review**

**Davisse-Paturet et al present an impressive analysis of data from a national cohort study, looking at the association between COVID-19 and subsequent suicidal ideation. This review considers the use of statistics in the paper.**

**The basic method of deriving models to predict the propensity of having COVID-19 illness or SARS-Cov-2 infection, and using these to weight the analysis of each exposure variable and the outcome, is good. I thought the paper was clearly written, and the results are well presented and interpreted, so my comments are fairly minor.**

*Response:*

We sincerely thank Alex McConnachie for the in-depth statistical review of our manuscript.

**One exception is the use of the E-value. Whilst I think it is a nice idea, the more I read about it, the less convinced I am that it really adds very much. Ultimately, it is simply a transformation of the estimated association between exposure and outcome, so I think it could be excluded without affecting the paper.**

We understand the statistical reviewer’s point of view. We initially added the E-value analysis to explain the reliability of our findings in a different way. Based on this comment and as four sensibility analyses were added to the revised manuscript, the E-value paragraphs were removed from the main revised text and added in supplementary material.

**Lines 184-188 describe the methods for dealing with missing covariate data. Personally, I think if you are going to that much trouble, it is worth doing multiple imputation of the missing data, so as to allow for the additional uncertainty due to the missingness.**

We agree with the reviewer and performed the suggested multiple imputation.

Page 10 Line 246-249: “All the analyses were performed using SAS V9.4. Tests were two-sided and considered statistically significant at p < 0.05. Missing data on covariates (up to 4.84%) were handled using the fully conditional specification method and assuming that data were missing at random (SAS MI procedure, FCS statement).”

**Lines 213-214 say that the estimated association between COVID-19 illness and suicidal ideation was unchanged after IPW. I would disagree. The estimate reduces from 1.90 to 1.43, so it had roughly halved after reweighting. In Figure 2, there is a visible reduction in the estimated association. The authors describe the adjusted association of 1.43 as "an almost 1.5 higher risk", which I think is a slight exaggeration. I would just say "a 43% higher risk".**

We completely agree with the reviewer and changed the relevant sentences.

Page 11 Line 263-265: “As shown in Fig 3, after IPWeighting, self-reported COVID-19 like symptoms in 2020 were associated with a higher risk of suicidal ideation in 2021 (RRipw: 1.43 [1.20 – 1.69]) while serology-confirmed SARS-CoV2 infection were not (RRipw: 0.89 [0.70 – 1.13]).”

**Did the authors consider a combined model, adjusting for both COVID-19 illness and SARS-Cov-2 infection? Was there a big overlap between the two? Could there be an interaction?**

*Response*:

Among participants who reported COVID-19 like symptoms, 30.8% [29.4; 32.2] had a serology-confirmed SARS-CoV2 infection. Among participants who reported no COVID-19 like symptoms, 6.3% [6.0- 6.6] had a serology-confirmed SARS-CoV2 infection. The question of the interaction between serology-confirmed infection and self-reported COVID-19 like symptoms was explored using sensitivity subgroup analyses as explained above in the response to the question 6) raised by the academic editor.

**Line 234: These sorts of analysis have come to be known as "causal" analyses, but I think this is a misleading term. These methods are a good way to adjust an analysis of the association between an exposure and an outcome, but the final estimated association cannot be described as "causal". As with any observational study, the possibility of unobserved confounders will always remain.**

*Response*:

We completely agree with the reviewer and the revised manuscript is more tempered when it comes to causality language.

**In Figure 1, the final set of exclusions are described as having "Insufficient 'Do not wish to answer' to be analysed". Is the word "Insufficient" correct here?**

Response:

The following changes were made.

Fig 2 (ex-Fig 1): ““do not wish to answer” modalities (n=105)”

Page 8 Line 181: “Do not wish to answer modalities had to be removed because of small sample sizes.”

**I would rather see Table 1 with separate columns for those with and without the outcome, and for those who are exposed/unexposed. The "by-outcome" table is given in table S2, but could "by-exposure" tables also be shown?**

*Response*:

The requested changes were made and the by-outcome table, as well as by-exposure tables, were added to the main text.

**Finally, and I hope this is just a typo, but in the supplement, the weights for the exposed group are given as 1/(1-p), and for the unexposed as 1/p. I believe it should be the other way round. Those who were exposed, despite having a low probability of being exposed (i.e. small p) should be up-weighted in the analysis, as should those who were unexposed, despite a high probability of being exposed (high p, small 1-p).**

We thank the reviewer for its well-informed eye. After reading the comment, we double checked the scripts and can now confirm that this was a typo.

**Also, I prefer "exposed/unexposed" over "case/control" in this context, since this is not a case-control study.**

We completely agree with the reviewer and made the requested changes.

**Reviewer #2: I read the manuscript "COVID-19 illness, SARS-CoV2 infection, and subsequent suicidal ideation in the French nationwide population-based EpiCov cohort: a propensity score analysis of more than 50,000 individuals" with great interest and would like to share the following few thoughts with the authors.**

**1. The authors found that self-reported COVID-19 symptoms are indicative of suicidal ideation whereas serology-confirmed SARS-CoV2 infection was not. Before we speculate the underlying reasons for such disparity, I wonder if the authors could clarify the number of participants with both self-reported symptoms and serology-confirmed infection. Namely, among the 9.57% with a SARS-CoV2 infection in 2020 and 13.23% that reported COVID-19 symptoms, how many had both?**

**2. Related to this, can the authors provide a comparison between the above two groups, something similar as the Supplementary table 2? This will likely help us to understand the differences between these two groups of participants.**

*Response to point 1 and 2*:

Among participants who reported COVID-19 like symptoms, 30.8% [29.4; 32.2] had a serology-confirmed SARS-CoV2 infection. Among participants with a serology-confirmed SARS-CoV2 infection, 42.6% [40.8; 44.4] reported COVID-19 like symptoms. The suggested tables are now included in the main text (tables 2 and 3). Moreover, as also suggested by the other reviewers, we added sensitivity analyses exploring association of self-reported COVID-19 like symptoms in 2020 with subsequent suicidal ideation in 2021 separately in the serology-confirmed SARS-CoV2 infection group and the group with negative serologies.

Page 10 Line242-245: “Lastly, to explore the intersection of both COVID-19 exposures with respect to suicidal ideation, association of self-reported COVID-19 like symptoms before December 2020 with subsequent suicidal ideation in 2021 was separately assessed according the SARS-CoV2 serological status.”

Page 11 Line 287-291: “Lastly, in the serology-confirmed SARS-CoV2 infection group, self-reported COVID-19 like symptoms in 2020 were not associated with suicidal ideation in 2021 (RR_ipw_: 1.49 [0.95 – 2.32]), while in the negative serology group, self-reported COVID-19 like symptoms before December 2020 were associated to a higher risk of subsequent suicidal ideation in 2021 (RR_ipw_: 1.49 [1.22 – 1.83]).”

**3. According to Supplementary Table 1, the participants included in the final analysis are rather different from the participants excluded from the analysis. Specifically, there seems to be a higher prevalence of self-reported COVID-19 symptoms, but a lower prevalence of suicidal ideation, among the included participants, compared with excluded participations. This will likely lead to an underestimated association between self-reported symptoms and suicidal ideation. This is however not the case for serology-determined infection.**

*Response:*

We thank the reviewer for this relevant point and have included it in the discussion section.

Page 14-Line 385-388: “. Of note, included participants were less likely to report suicidal ideation in 2021 but more likely to report COVID-19 like symptoms, potentially leading to an underestimation of the association between the two. Such difference were however not found for serology-confirmed SARS-CoV2 infection.”

**4. Related to the above, I think the authors cannot claim their data as population-based, because the study participants included in the analyses are not representative of the underlying source population. This should rather be discussed as a limitation of the study.**

*Response*:

We agree, our study in not representative. When using the word nationwide, we meant that the study included both mainland France as well as oversea territories. The word nationwide has been removed from the revised manuscript. Moreover, the word “population-based” when referring to the EpiCov was indeed misused and is now replaced by “general population”. As suggested, discussion of limited generalizability is now highlighted in the abstract as well as discussion section. Of note, descriptive statistics can be considered as representative of the general population as they were weighted by study weights.

Abstract – method section: “Limitations of this study include the use of a single question to assess suicidal ideation, the use of self-reported history of mental health disorders, and limited generalizability due to attrition bias.”

Page 14-Line 382-388: “Lastly, although the EpiCov cohort is based on a randomly selected sample, attrition overtime as well as successive steps of participant selection for analyses purpose limit the generalizability of our results. Difference between included and excluded participants can be found in supplementary table 1. Of note, included participants were less likely to report suicidal ideation in 2021 but more likely to report COVID-19 like symptoms, potentially leading to an underestimation of the association between the two. Such difference were however not found for serology-confirmed SARS-CoV2 infection.”

**Reviewer #3: This manuscript examines the impact of COVID-19 illness and SARS-CoV2 infection on suicidal ideation in a cohort, EpiCOV, which is a longitudinal nationwide French cohort aimed at examining the impact of the pandemic on daily life and health of individuals. The study consisted of three waves of data collection. The sample size analyzed was 52,050 participants aged 15 years and older who had participated in the second wave of follow-up and participated in dried-blood-spot sampling kit to test for antibodies against SARS-COV2's spike protein S1 domain and answered questions regarding suicidal ideation. The survey asked the following question at the 3rd wave of the study: "Since December 2020, have you thought about committing suicide (yes/no)?". There were also questions about thoughts of suicide in the last 12 months and lifetime suicide attempts. Self-reported symptoms of COVID-19 were assessed and defined as a self-report of any unusual episode of sudden loss of taste/smell or any unusual episode of fever alongside a cough, shortness of breath, or chest oppression. The study is timely and addresses an important research question that has not been studied yet. The large sample size, longitudinal design, examining serology to test for COVID-19 antibodies, and the use of propensity scores to control for covariates and potential confounders are all major strengths. Below are some concerns that if addressed, it would strengthen the study.**

**- The manuscript is not clear on the temporality of assessments between exposure and outcome. It would help to include a figure to describe the waves of the study and when questions were asked about exposure and outcome. The outcome is described at multiple points in the manuscript with questions about suicidal ideation since the pandemic and in the last 12 months.**

*Response*:

We agree that this aspect could be better clarified. The Fig describing the follow-up of the EpiCov cohort has been moved from the supplementary material to the main revised text (Fig 1).

**- The authors do not describe the baseline characteristics of the sample analyzed vis-a-vis the rest of the sample who were not included in the analyses. It is difficult to determine potential ascertainment biases without this comparison.**

*Response*:

An included/excluded comparison is available in supplementary material table 1. Very briefly, as expected with such sample size, almost variable were statistically significant between the included and excluded groups. Of note, excluded participants were more likely to report suicidal ideation but less likely to report COVID-19 like symptoms which could have underestimated our findings.

Page 14-Line 382-388: “Lastly, although the EpiCov cohort is based on a randomly selected sample, attrition overtime as well as successive steps of participant selection for analyses purpose limit the generalizability of our results. Difference between included and excluded participants can be found in supplementary table 1. Of note, included participants were less likely to report suicidal ideation in 2021 but more likely to report COVID-19 like symptoms, potentially leading to an underestimation of the association between the two. Such difference were however not found for serology-confirmed SARS-CoV2 infection.”

**- It is not clear how the authors assessed prior history of suicidal behavior and mental disorders. It would help to include more details and whether there were screens for depression and anxiety symptoms.**

*Response*:

We agree the pre-pandemic mental health disorder covariate was not fully detailed in the main text nor the supplementary material. As the main text is already quite long, complete information regarding this covariate has been added in supplementary material

Supplementary material, first section: “At baseline in May, participants were asked to report if they had any pre-pandemic chronic condition or limiting health condition that lasted at least 6 months. If so, they were then asked to select which ones from a list which included depression, anxiety and mental impairment. Participants reporting at least one of these three conditions were included in the pre-pandemic mental health disorder group.

At the first follow-up in Autumn 2020, participants were asked to report life-course suicide attempt and if so, the period of the last one, i.e. before Autumn 2019, between Autumn 2019 and 17th of March 2020, between 17th of March and the 11th of May 2020 (first lockdown), between 12th of May 2020 and Autumn 2020. Participants reporting a last suicide attempt before Autumn 2019 were included in the pre-pandemic mental disorder group.

At the second follow-up in Summer 2021, participants were asked if a physician ever told them they had a mental health or substance use disorder. If so, they were asked to report which one and when (before the pandemic vs since the pandemic) from a list. The list included anxiety disorders, mood disorders, bipolar disorders, eating disorders, personality disorders, substance use disorders and schizophrenia. Participants reporting a physician ever telling them they had at least one of these disorders before the pandemic were included in the pre-pandemic mental health disorder group.”

**- It is not clear why the authors excluded those with prior history of suicidal behavior between November 2019 and 2020 when someone could reattempt and those with a prior history are at highest risk for re-attempt.**

*Response*:

We agree with the reviewer and revised the relevant paragraph.

Page 6 Line 166-180: Suicidal ideation in 2020, as well as life course suicide attempts were assessed at the first follow-up in Autumn 2020 (Fig 1). The related questions were respectively: “In the last 12 months, have you thought about “killing yourself by suicide”?” and “In your lifetime, have you ever attempted suicide? If so, when was the last one?”. Yet, mediators are not supposed to be included in propensity score calculation. Suicidal ideation and suicide attempts in 2020 could act as mediator in the association of self-reported COVID-19 like symptoms in 2020 and serology-confirmed SARS-CoV2 infection in 2020 with suicidal ideation in 2021. But, in EpiCov, no data were available regarding the first occurrence of COVID-19 like symptoms in 2020 or date of SARS-CoV2 infection in 2020. We could therefore not ascertained if suicidal ideation or suicide attempt in 2020 occurred before or after the two COVID-19 exposures. To ensure no mediator was included in propensity score calculation, participants reporting suicidal ideation or a history of suicide attempt in 2020 or who did not provide information on the timing of their last suicide attempt, had to be removed from the statistical analyses. Participants who reported a last suicide attempt before 2020 were included in the pre-pandemic mental health disorder covariate.”

**- It would be important to include more details about the study weights and how they were computed.**

The way in which study weights were computed is thoroughly described in: Warszawski J, Bajos N, Barlet M, de Lamballerie X, Rahib D, Lydié N, et al. A national mixed-mode seroprevalence random population-based cohort on SARS-CoV-2 epidemic in France: the socio-epidemiological EpiCov study. medRxiv. 2021:2021.02.24.21252316). As study weights were only used for descriptive purpose in our study and as the methodology section of our manuscript is quite dense, we think that adding the description of study weights computation could be confusing for readers who might get lost between the study weights and the propensity scores weights. Nonetheless, should the reviewer and academic team think that the study weight methodology should be explained, we can add it in the supplementary material.

**- The description of the sample's characteristics is based on some subgroups when there's variability (e.g., non-smokers when there were a good proportion of the sample who were past or current smokers).**

*Response*:

We are sorry, we did not understand what was requested by the reviewer here. With some more details, we will certainly be able to clarify in the manuscript the point raised here.

**- One of the limitations of the study is the assessment of ideation and attempt using individual items. The questions do not differentiate between passive and active suicidal ideation; and whether the suicide attempts were actual vs. gestures and/or preparatory behaviors. The authors need to refer to suicide attempt as suicidal behavior as the latter is often referring to actual suicide attempt, which was not assessed here.**

*Response*:

We agree with the reviewer the use of a single question to assess suicidal ideation is not ideal. Moreover, the difference between active and passive suicidal ideation was not collected. We have added a paragraph in the discussion’s limitation section.

Page 14 Line 371-376: “A second limitation of our study is the use of a single question to assess suicidal ideation, which did not specifically explore whether suicidal ideation was active or passive, or whether the participants had an explicit suicidal plan. Scales specifically designed to assess suicidal ideation and behaviors (31-33) would have given a more accurate picture of the severity, chronicity and intentionality of these thoughts and their association with COVID-19 like symptoms.”

***Reference***

1.	Sterne JA, White IR, Carlin JB, Spratt M, Royston P, Kenward MG, et al. Multiple imputation for missing data in epidemiological and clinical research: potential and pitfalls. Bmj. 2009;338:b2393.

2.	Li P, Stuart EA, Allison DB. Multiple Imputation: A Flexible Tool for Handling Missing Data. Jama. 2015;314(18):1966-7.

3.	Hughes RA, Heron J, Sterne JAC, Tilling K. Accounting for missing data in statistical analyses: multiple imputation is not always the answer. Int J Epidemiol. 2019;48(4):1294-304.

4.	Kontopantelis E, White IR, Sperrin M, Buchan I. Outcome-sensitive multiple imputation: a simulation study. BMC Med Res Methodol. 2017;17(1):2.
