# Supplementary material for: Suicidal ideation following self-reported COVID-19-like symptoms or serology-confirmed SARS-CoV-2 infection in France: A propensity score weighted analysis from a cohort study
Source: PLoS Med. 2023 Feb 14;20(2):e1004171. doi: 10.1371/journal.pmed.1004171 (PMC10072374; doi:10.1371/journal.pmed.1004171)
Supplement: Attachment — Submitted filename: Response to reviewer.docx [file pmed.1004171.s020.docx]

Camille Davisse-Paturet, on behalf of all co-authors
CESP-INSERM U1018
Equipe Psychiatrie du Développement et trajectoires,
97 boulevard de Port-Royal,
75014 Paris, France
[Camille.davisse-paturet@inserm.fr](mailto:Camille.davisse-paturet@inserm.fr)
+33158412848

1^st^ December, 2022

Dear Reviewers,

We thank you for the time you took to thoroughly read and comment our work once more.

Below is our point by point response to each comment, the changes made and where to find them in the revised version.

We hope that you will find our revision satisfactory and remain at your disposal if needed.

Respectfully,

Camille Davisse-Paturet, on behalf of all co-authors

**Requests from Editor:**

**Please address the comment from the statistical reviewer.**

**The Data Availability Statement (DAS) requires revision. For each data source used in your study:**

**b) If the data are owned by a third party but freely available upon request, please note this and state the owner of the data set and contact information for data requests (web or email address).**

We hope the following DAS statement to be more clear:

All anonymous aggregated data concerning the results presented in this paper are available online and in the supporting tables and the supporting information files. The EpiCov dataset can be made available for research purpose after submission to approval of French Ethics and Regulatory Committee procedure (Comite´ du Secret Statistique, CESREES and CNIL). Access procedure is available on CASD (<https://www.casd.eu/>). Additional information can be addressed to the corresponding author.

**Please correct 'SARS-CoV2' to 'SARS-CoV-2' throughout.**

The requested changes have been made.

**Please structure your abstract using the PLOS Medicine headings (Background, Methods and Findings, Conclusions). Please combine the Methods and Findings sections into one section, “Methods and findings”.**

**Please update the beginning of your abstract to read: 'A higher risk of suicidal ideation associated with self-reported COVID-19 like symptoms or COVID-19 infection has been observed in cross sectional studies, but evidence from longitudinal studies remains limited. This study's aims...'**

**In the last sentence of the Abstract Methods and Findings section, please describe the main limitations of the study's methodology (the sentence that currently finishes the abstract 'Methods' can be relocated).**

**In the Abstract Conclusions, please avoid vague statements such as those in the concluding sentence of the abstract. Instead, mention specific implications substantiated by the results.**

**Please include a basic demographic summary of your sample in the Methods and Findings of your Abstract.**

All requested changes regarding the abstract have been made.

The conclusion now reads: ”If self-reported COVID-19 like symptoms were associated with later suicidal ideation, the exact role of SARS-CoV-2 infection with respect to suicide risk has yet to be clarified. Including mental health resources in COVID-19 related settings could encourage symptomatic individuals to care for their mental health and limit suicidal ideation to emerge or worsen. In France, these resources could for example be included in the prevention messages explaining how to process when experiencing symptoms, or displayed in family doctors waiting room, pharmacies, or COVID-19 screening places. Mental health resources may be help lines numbers and websites, brief and accessible description of deteriorated mental health symptoms, or guidance on how to access fully covered psychologist appointments.”

The following demographic summary were added in the Method and Finding section: “Among 52,050 participants from the EpiCov cohort, 53.84% reported being a woman, 60.92% were over 45 years old, 82.01% were born in mainland France from parents born in mainland France and 59.38% completed high school.”

**Please structure your author summary using 2-3 single sentence bullet points for each of the three questions. Bullet points should be objective, brief, succinct, specific, accurate, and avoid technical language.**

The non-technical author summary has been rewritten as follow:

“Why was this study done?

There is a need to investigate suicide-related outcomes in individuals exposed to virus responsible for epidemics.

COVID-19 infection seems associated to a higher risk of suicidal ideation, but studies exploring the association over time are limited.

What did the researchers do and find?

We explored if COVID-19 like symptoms in 2020, as reported by participants, and SARS-CoV-2 infection in 2020, as confirmed by serological tests, were associated to a higher risk of suicidal ideation in 2021 in 52,050 participants followed three times from May 2020 and to the Summer 2021 in France.

Among these participants, we found that reporting COVID-19 like symptoms in 2020, but not having a serologically confirmed SARS-CoV-2 infection, was associated with a higher risk of reporting suicidal ideation in 2021. These results account for sociodemographic and health-related factors.

What do these findings mean?

Individuals experiencing COVID-19 like symptoms in the first year of the pandemic, even with no serological confirmation of the infection, were at higher risk of later suicidal ideation.

From a public health perspective, short communication on what to do when someone is experiencing distress, whether directly after the onset of symptoms or a few months later, may be beneficial to address increase in suicidal ideation.”

**Line 46: 'dose-response fashion'.**

As requested, the term “dose-response way” in the main text introduction section has been replaced by “dose-response fashion”.

**The terms gender and sex are not interchangeable (as discussed in https://www.who.int/health-topics/gender); please consider whether sex (and thus male/female) would be the more appropriate term dependent on how this covariate data was collected.**

As explained in our previous letter, the question was “Are you?” with possible answer being “A man, a woman, do not wish to answer”. We believe this to be closer to the WHO definition of gender rather than the definition of sex. But, as we are well aware of the importance of appropriate terms, we are ready to change the term to “sex” if the editors think it is more accurate. Of note, we screened both the main text and supplementary materials to make sure the term “sex” was note accidentally used instead of “gender”. The term “sexe” in the supplementary material is simply the French label of the variable used for gender.

**Please cite your Supporting Information as outlined here: https://journals.plos.org/plosmedicine/s/supporting-information**

The requested changes have been made.

**For international readers unfamiliar with how residential areas in France were affected during the pandemic, it may be useful to provide further details regarding this categorisation (i.e., brief overview of Grand-Est, Hauts-de-France-Ile-de-France).**

Departmental mortality rates for mainland France during the first COVID-19 epidemic wave in 2020 were added as supporting information (S2 supporting information) in the supplementary material word file.

**Line 226: 'sensitivity analysis'**

The requested change was made.

**Lines 229-241: This reads as a description of the sensitivity analysis results. Please only describe methodology in the Methods section and relocate description of findings to the Results section.**

The requested changes were made.

**Please update your STROBE checklist to use section names and paragraph numbers, as page/line numbers are likely to change.**

The STROBE checklist has been revised accordingly.

**Table 1 does not appear to be cited anywhere in the main text.**

The following citation has been added in the Method – Statistical analyses – Descriptive statistics section: “Study weights were applied to all descriptive statistics (Tables 1, 2, and 3) in order to take EpiCov’s design and attrition bias into account.”

**Please present numerators and denominators for percentages, at least in the Tables.**

As the reported percentages are weighted using study weights, division of numerators by denominators won’t reflect the percentages included in the tables. Moreover, the presented percentages are pooled percentages coming from five imputed data sets so sizes in numerators and denominators may vary from one data set to another. Yet, in order to sill give some information regarding numerators and denominators while limiting confusion, numerators and denominators of each descriptive table are now detailed in separate tables in the supporting tables file (S2, S3, S4, S5 Tables). The main text has been updated as follow:

Method section, descriptive statistics part :” Study weights were applied to all descriptive statistics (Tables 1, 2, 3, S1) in order to take EpiCov’s design and attrition bias into account. Briefly, the study weights accounted for demographic and socioeconomic indicators potentially linked to response probability and made available by INSEE from the tax data base. Study weights were also calibrated on margins of the general population according to census data and population projections [23]. The numerators and denominators for each descriptive table are available in the supporting tables file (S2, S3, S4, S5 tables). Because descriptive statistics were weighted by study weights the presented percentages do not reflect the division of numerators by denominators. Except for included/excluded comparison, all presented percentages were pooled from the five imputed data sets (more details below, mi procedure in SAS statistical software).”

**Please define the abbreviation DOM in Table legends.**

DOM stands for “Départements d’Outre-Mer”. But, as oversea territories was used throughout the main text we replaced “DOM” by “oversea territories”.

**The term 'mental health behaviour' may present a translation issue as the meaning is ambiguous. Please provide a definition of this term.**

The terms “mental health behaviour” have been replaced by the terms “mental health disorder” in all tables.

**Please update 'associated to' to 'associated with' throughout.**

The requested changes were made**.**

**Please consider tabulating the results of your sensitivity analyses and presenting these in the Supporting Information.**

Following the editor’s comment,; a figure (S5 figure) has been added to the supplementary material to illustrate results from the sensitivity analyses. We opted for a figure instead of the suggested table for homogeneity purpose, as results of the main analysis are presented as a figure.

**Lines 359-361: I feel this should be in the Limitations section.**

As the lines have changed we are not sure which sentence are referred to in this suggestion. As the last paragraph of the self-reported COVID-19 like symptoms and suicidal ideation part of the discussion section has been changed following the statistical reviewer comment, we hope the revised version also address the comment.

**Your concluding sentence (line 395-396) introduces the concept of socioeconomic involvement, despite this not being a key finding of your study. I would suggest moving this sentence earlier in the Discussion.**

The suggested change has been made. The following sentence has been added in the discussion section on self-reported COVID-19 like symptoms : “A possible way of preventing symptomatic individuals to experience suicidal ideation could therefore be through policies aiming at mitigating the socioeconomical impact of the pandemic [30].”

The conclusion section now reads: “… In a pandemic context, bringing awareness on mental health in the first few months following a symptom onset could limit an increase in suicidal ideation. Mental health resources could, for example, be made available in COVID-19-related settings such as prevention messages explaining how to process when experiencing symptoms, family doctors waiting room, pharmacies, or COVID-19 screening places. Example of mental health resources includes help lines numbers and websites, or brief and accessible description of what a deteriorated mental health can look like, and guidance on what to do when experiencing it.”

**Please carefully check your references for formatting issues, I noted problems with references 5, 12, and 22.**

**Reference 23 is a preprint and ought to be identified as such (see https://journals.plos.org/plosmedicine/s/submission-guidelines#loc-references for further guidance).**

The requested changes have been made.

**Comments from Reviewers:**

**Reviewer #1: Alex McConnachie, Statistical Review**

**I thank Davisse-Paturet et al for their responses to my original comments, and I am mostly happy with these.**

**The one exception is their handling of the association between COVID-19 illness and suicidal ideation separately in those with and without confirmed SARS-Cov-2 infection. The authors state that there was an association in the uninfected subgroup, but not the infected subgroup. However, the RRs are identical in the two groups. The fact that the p-value is significant for one but not the other is not the correct way to assess these associations. It would be better to fit a single model and test the interaction between illness and infection; given the results presented, this will be non-significant, meaning that there is no evidence to suggest that the association between COVID-19 illness and suicidal ideation is any different in those with or without confirmed infection.**

We thank statistical reviewer Alex McConnachie for taking, once again, the time to read our paper. Regarding the interaction between COVID-19 symptoms and SARS-CoV-2 serology, we agree the relative risks are similar and the lack of significance in the serology-confirmed SARS-CoV-2 infection group is likely to be due to lack of statistical power. The manuscript has been revised accordingly.

In exploratory analyses, we assessed the interaction of serology-confirmed SARS-CoV-2 infection in the association of COVID-19 like symptoms with later suicidal ideation. To do so, we tested the interaction in a modified Poisson regression model with suicidal ideation as the outcome and both the COVID-19 exposures as well as the interaction term as explanatory variables. The interaction was indeed non-significant. We proceeded this way because we were unsure that including an interaction term in the modified Poisson regression model while using inverse probability weighting was methodologically sound. If the statistical reviewer has any references to share on this topic, we would happily read them.

The main text now reads:

Last paragraph in the sensitivity analyses part of the results section: “Lastly, the sensitivity analysis exploring the association of self-reported COVID-19 like symptoms with later suicidal ideation according to SARS-CoV-2 serology yielded similar results, although statistical significance was reached in one group but not the other (RR_ipw_: 1.49 [0.95 – 2.32]), in the serology-confirmed SARS-CoV-2 infection group; RR_ipw_: 1.49 [1.22 – 1.83], in the negative SARS-CoV-2 serology group) (S5 figure).

Last paragraph of the self-reported COVID-19 like symptoms and suicidal ideation part of the discussion section: “Difference seen in statistical significance for the association of COVID-19 like symptoms with later suicidal ideation according to SARS-CoV-2 serological status is likely to be due a lack of statistical power to assess such association in the serology-confirmed SARS-CoV-2 infection group.”

**Reviewer #2: Thank you for the responses to my comments.**

We thank Reviewer #2 once again for taking the time to review our paper and are pleased our responses answered their comments.
